# PFO-Mediated Paradoxical Coronary Embolism Causing Myocardial Infarction: Concordant Imaging Findings

**DOI:** 10.1016/j.jaccas.2026.109665

**Published:** 2026-08-18

**Authors:** Anupam Shrestha, Chiew Wong, James Theuerle, Rifly Rafiudeen

**Affiliations:** a Department of Medicine, University of Melbourne, Melbourne, Victoria, Australia; b Department of Cardiology, Northern Health, Melbourne, Victoria, Australia; c Department of Cardiology, Austin Health, Melbourne, Victoria, Australia

**Keywords:** acute coronary syndrome, anticoagulation, bubble echocardiography, cardiac magnetic resonance, chest pain, coronary angiography, echocardiography, myocardial infarction, thrombosis, thrombus

## Abstract

**Background:**

Paradoxical coronary embolism is a rare cause of myocardial infarction in which establishing causality may be challenging without an identifiable venous thromboembolic source.

**Case Summary:**

A 36-year-old man presented with chest pain and elevated troponin. Coronary angiography demonstrated distal thrombotic occlusion of the right posterolateral ventricular branch within an otherwise smooth coronary tree without significant atherosclerosis. Cardiac magnetic resonance showed subendocardial-to-transmural infarction with edema confined to the corresponding vascular territory. Transthoracic and transesophageal echocardiography demonstrated a patent foramen ovale (PFO) with right-to-left shunting, and no alternative embolic source was identified. Findings supported probable PFO-mediated paradoxical coronary embolism, and percutaneous closure was performed.

**Discussion:**

Concordant angiographic and cardiac magnetic resonance findings, together with echocardiographic demonstration of right-to-left shunting, supported probable paradoxical coronary embolism and guided conservative acute management followed by PFO closure.

**Take-Home Messages:**

Plaque-free distal coronary thrombus should prompt consideration of embolism. Multimodality imaging can support mechanism-informed diagnosis and secondary prevention.

## Graphical Abstract

**Figure:**
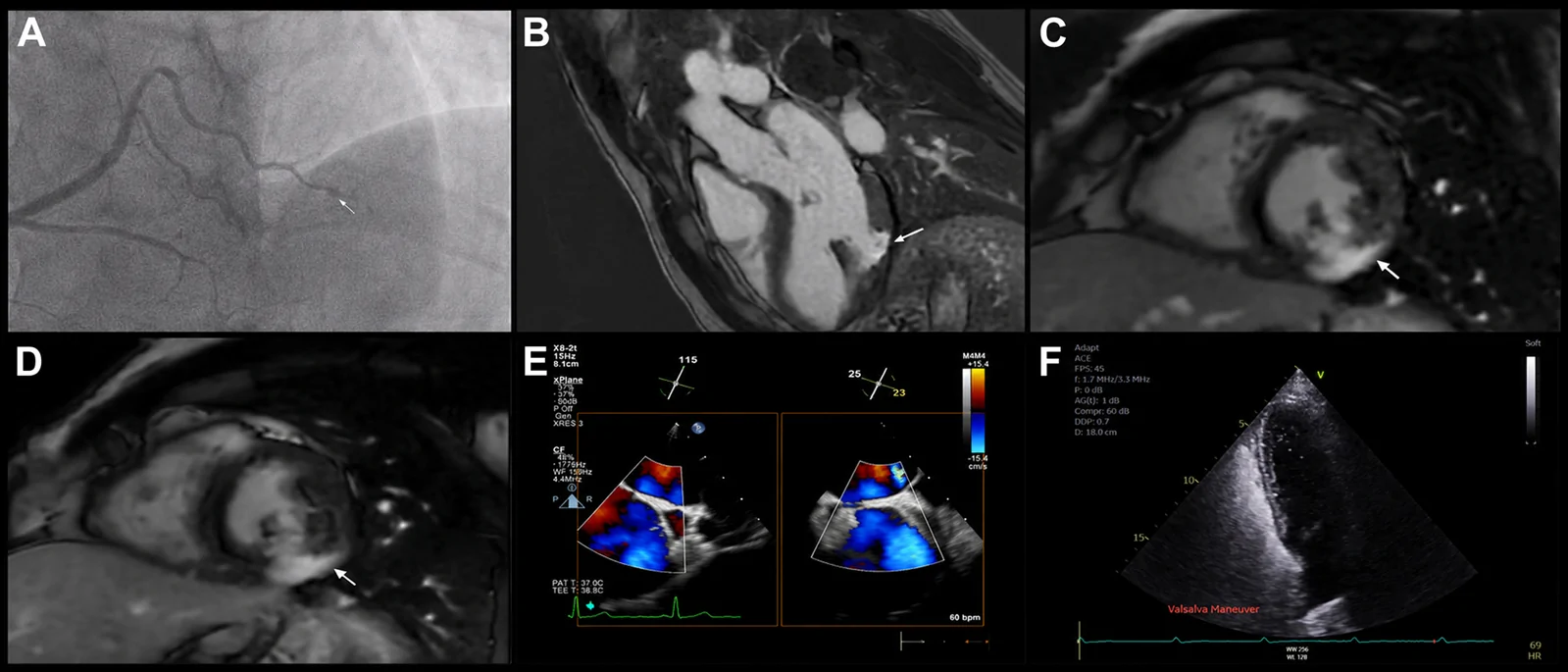

## History of Presentation

A 36-year-old man presented with sudden-onset epigastric and left-sided chest pain radiating to the left arm, with associated diaphoresis and dizziness. He reported no dyspnea, pleuritic symptoms, or preceding Valsalva-type maneuver, including heavy lifting, straining, coughing, or breath holding. His symptoms were not relieved by sublingual nitroglycerin. On examination, he was normotensive, with normal first and second heart sounds and no added sounds or murmurs.

**Take-Home Messages**

• In patients with myocardial infarction and plaque-free coronary arteries on angiography, a distal thrombus or abrupt vessel cut-off should prompt consideration of coronary embolism and targeted evaluation for an embolic source.
• Concordance between distal coronary thrombus on angiography and cardiac magnetic resonance-confirmed infarction in the corresponding vascular territory, together with echocardiographic demonstration of right-to-left shunting through a patent foramen ovale, can support a mechanism-based diagnosis of probable paradoxical coronary embolism and guide secondary prevention, including consideration of percutaneous patent foramen ovale closure in selected patients.

## Past Medical History

The patient reported an atrial septal defect diagnosed in infancy that was thought to have closed spontaneously by early adulthood. A transthoracic echocardiogram performed before the index presentation demonstrated normal left ventricular size and systolic function. On that study, no residual atrial septal defect was identified on color Doppler, although an agitated saline contrast study was not performed. This echocardiogram also showed suboptimal pulmonary valve visualization, with Doppler findings consistent with mild pulmonary stenosis and trivial pulmonary regurgitation. Additional medical history included gastroesophageal reflux disease and depression. He smoked socially and had no personal or family history of thromboembolic disease.

## Differential Diagnosis

The initial differential diagnosis included acute coronary syndrome, pulmonary embolism, myocarditis, pericarditis, stress-induced cardiomyopathy, and aortic dissection. Noncardiac causes, including musculoskeletal chest pain, gastroesophageal reflux disease, and esophageal spasm, were also considered at presentation.

## Investigations

The key multimodality imaging findings are shown in Figure 1. Electrocardiography demonstrated sinus rhythm without diagnostic ischemic changes (Figure 2). High-sensitivity troponin peaked at 15,019 ng/L. Lipid profile, glycated hemoglobin, and inflammatory markers were all within normal limits.

**Figure 1:**
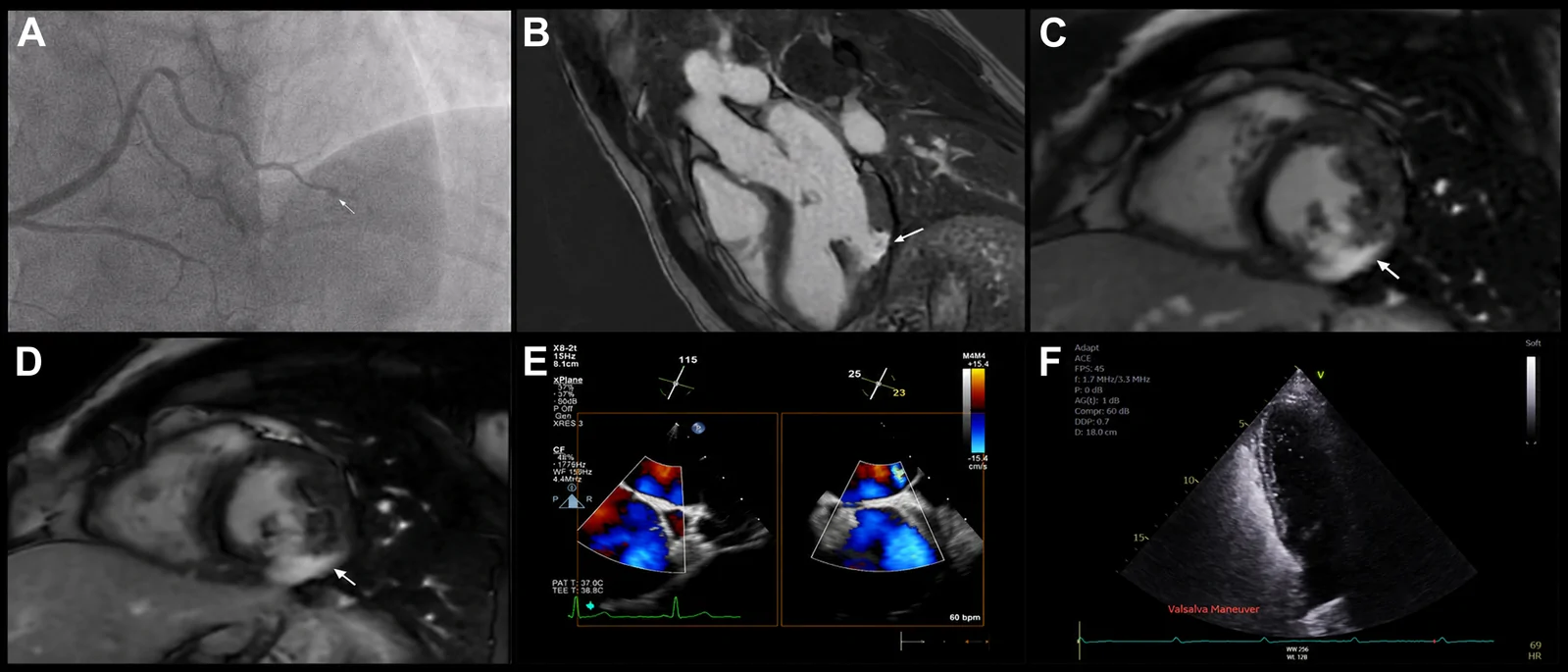
Multimodality Imaging Findings Supporting Paradoxical Coronary Embolism Through a Patent Foramen Ovale (A) Coronary angiography demonstrates thrombus in the distal right posterolateral ventricular branch, with otherwise angiographically smooth coronary arteries and no evidence of atherosclerotic plaque. (B) Cardiac magnetic resonance long-axis late gadolinium enhancement imaging demonstrating infarction in the posterolateral territory. (C) Short-axis late gadolinium enhancement imaging demonstrating transmural infarction in the corresponding posterolateral territory. (D) T2-weighted fat-suppressed cardiac magnetic resonance sequence demonstrating myocardial edema, consistent with acute myocardial injury. (E) Transesophageal echocardiography (mid-esophageal bicaval view) with color Doppler demonstrating a patent foramen ovale and a focal jet across the interatrial septum. (F) Transthoracic echocardiography with microbubble contrast during Valsalva maneuver demonstrating early appearance of microbubbles within the left atrium, consistent with right-to-left interatrial shunting. Together, these findings demonstrate concordance between coronary thrombus localization, tissue-level infarction, and right-to-left shunting, supporting paradoxical coronary embolism. Arrows in (A to D) indicate the key angiographic and cardiac magnetic resonance findings. CF = coronary flow; PAT = pulmonary artery acceleration time; TEE = transesophageal echocardiography.

**Figure 2:**
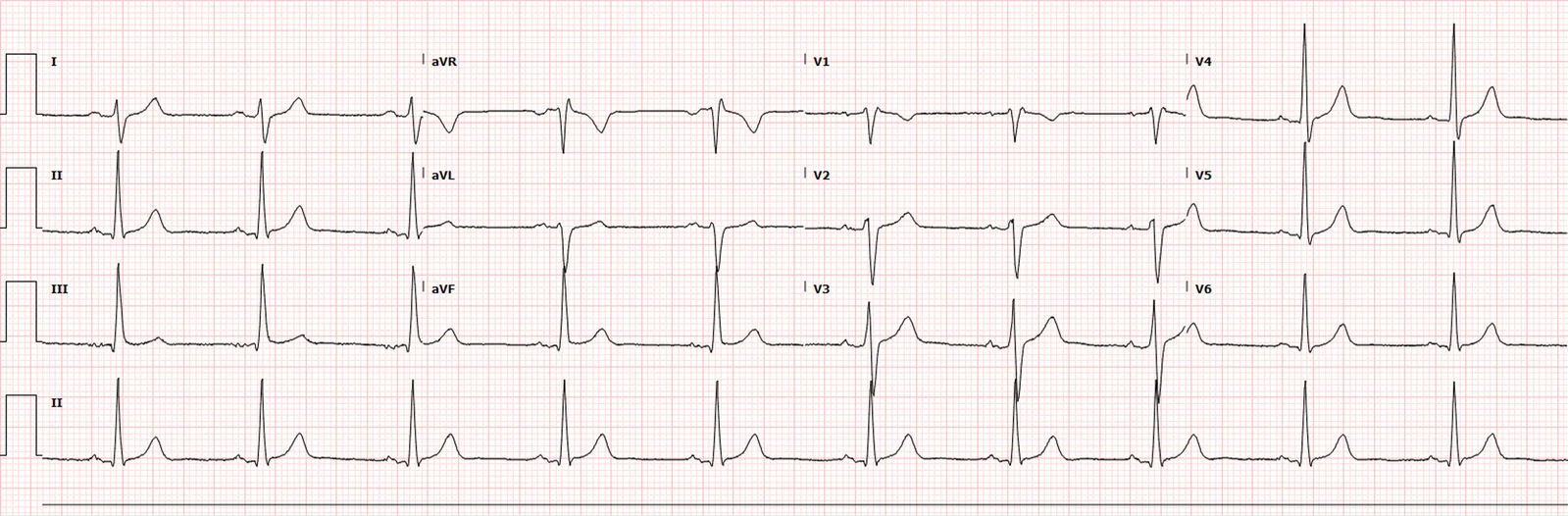
Admission Electrocardiogram Admission 12-lead electrocardiogram demonstrated normal sinus rhythm without diagnostic ischemic ST-segment or T-wave changes. aVF = augmented vector foot lead; aVL = augmented vector left arm lead; aVR = augmented vector right arm lead; V1-V6 = precordial leads V1 through V6.

Coronary angiography demonstrated a right-dominant circulation with otherwise angiographically smooth coronary arteries. A small distal filling defect was identified in the right posterolateral ventricular branch, consistent with a distal coronary thrombus causing abrupt vessel cut-off. No atherosclerotic plaque or stenosis was seen elsewhere. Ventriculography demonstrated mild inferoposterior hypokinesis. Intracoronary imaging with intravascular ultrasound or optical coherence tomography was not performed because the culprit abnormality was confined to a small-caliber distal branch, with no proximal stenosis or atherosclerotic lesion suitable for intravascular assessment or intervention.

Cardiac magnetic resonance (CMR) imaging demonstrated preserved left ventricular systolic function, with an ejection fraction of 55%, and focal hypokinesia of the mid-posterior segment. T2-weighted imaging showed myocardial edema, and late gadolinium enhancement revealed a subendocardial-to-transmural infarct pattern in the corresponding posterolateral vascular territory. No left ventricular thrombus was identified. Mild pulmonary stenosis, with a peak velocity of 2.0 m/s, poststenotic pulmonary trunk dilatation, and mild right atrial enlargement were also noted.

Transthoracic echocardiography demonstrated normal left ventricular size and systolic function, without intracardiac thrombus. An agitated saline contrast shunt study performed during Valsalva maneuver demonstrated early left atrial microbubble opacification, consistent with an intracardiac right-to-left shunt. Subsequent transesophageal echocardiography with color Doppler confirmed a patent foramen ovale (PFO), with no additional atrial septal defect or left atrial appendage thrombus identified. Intracardiac echocardiography was not performed.

Comprehensive thrombophilia and autoimmune screening were negative. Bilateral lower limb ultrasonography excluded deep vein thrombosis, whereas computed tomography pulmonary angiography showed no pulmonary embolism. Prolonged rhythm monitoring showed persistent sinus rhythm without atrial fibrillation.

## Management

The patient received initial treatment with dual antiplatelet therapy and therapeutic-dose enoxaparin before transitioning to apixaban. In the absence of an alternative embolic source, the presence of a right-to-left interatrial shunt supported probable paradoxical coronary embolism as the most likely mechanism of infarction. Following a multidisciplinary heart team discussion, percutaneous PFO closure was performed using a 25-mm PFO occluder without complication. Equipment used for the diagnostic and interventional procedures is summarized in Table 1.

## Outcome and Follow-Up

Follow-up imaging confirmed satisfactory device position with no residual interatrial shunt. At 1-year follow-up, anticoagulation was discontinued, and lifelong aspirin was continued.

## Discussion

Coronary embolism is an under-recognized cause of myocardial infarction. When angiography demonstrates a distal thrombotic occlusion within otherwise plaque-free coronary arteries, embolism should be considered, and evaluation should extend beyond confirming myocardial injury to clarifying its mechanism. In this context, CMR is particularly useful for distinguishing ischemic from nonischemic injury, defining acuity, and determining whether the infarct pattern corresponds to the angiographic abnormality.

In this case, a discrete distal thrombus in the right posterolateral ventricular branch, within an otherwise angiographically smooth coronary tree, favored embolism over plaque-mediated in situ thrombosis. The absence of diagnostic ischemic changes on the standard 12-lead electrocardiogram did not exclude infarction in this small distal territory, where electrical changes may be subtle or absent. CMR provided tissue-level confirmation of acute ischemic injury in the corresponding posterolateral territory, demonstrating subendocardial-to-transmural late gadolinium enhancement with associated T2-weighted myocardial edema. This concordance between angiography and CMR established ischemic myocardial injury as the cause of the troponin rise, excluded common mimics such as myocarditis and stress-induced cardiomyopathy, and prompted targeted evaluation for an embolic source.

No alternative embolic source was identified despite comprehensive investigation, including prolonged rhythm monitoring, thrombophilia screening, and bilateral lower limb color Doppler ultrasound. Transthoracic echocardiography with agitated saline contrast and subsequent transesophageal echocardiography demonstrated a PFO with right-to-left interatrial shunting, providing a plausible route for paradoxical embolism. Mild pulmonary stenosis with right atrial enlargement may have increased susceptibility to transient right-to-left flow across the PFO, although the stenosis was mild and its contribution to the index event remains uncertain. Importantly, the absence of an identifiable venous source does not exclude paradoxical embolism and is consistent with observations from PFO-associated stroke, where embolic sources are frequently transient or clinically silent and demonstrable in only a minority of cases.^1^

Using a recognized diagnostic framework for coronary embolism, this case is best classified as probable rather than definite coronary embolism.^2^ The angiographic finding fulfills one major criterion, whereas the PFO constitutes an embolic risk factor within the score. Although CMR falls outside the formal scoring system, it provided important corroborative evidence for the proposed diagnosis.

Paradoxical coronary embolism via a PFO has been reported in patients without angiographic evidence of coronary atherosclerosis. However, establishing the pathophysiologic sequence can remain challenging when key diagnostic elements are absent. In previous reports, either coronary thrombus was not directly visualized, or infarction was not confirmed at the tissue level in the corresponding vascular territory.^3^^,^^4^ This case adds to the literature by integrating direct visualization of a distal coronary thrombus, CMR-confirmed infarction in the same vascular territory, and echocardiographic demonstration of right-to-left interatrial shunting, thereby supporting a probable unifying mechanism of PFO-mediated paradoxical coronary embolism.

Given the distal location of the thrombus, acute management was conservative because the lesion involved a small-caliber branch, with preserved coronary flow elsewhere and no angiographic evidence of an underlying atherosclerotic target. In this context, aspiration thrombectomy or further instrumentation was considered unlikely to provide benefit and carried potential risks, including distal embolization and vessel injury. This approach was consistent with contemporary evidence that does not support routine thrombus aspiration. Therefore, antithrombotic therapy was used as the primary acute treatment strategy, addressing the thrombotic event while avoiding procedural harm.^5^

Evidence guiding PFO closure after coronary embolism remains limited, and randomized data specific to this population are lacking. However, trials in patients with cryptogenic stroke, including Randomised Evaluation of Recurrent Stroke Comparing PFO Closure to Established Current Standard of Care Treatment (RESPECT) and Patent Foramen Ovale Closure or Anticoagulants versus Antiplatelet Therapy to Prevent Stroke Recurrence (CLOSE), demonstrated sustained reduction in recurrent cerebrovascular events following percutaneous PFO closure compared with medical therapy.^6^^,^^7^ Although these findings cannot be directly extrapolated to myocardial infarction, they support consideration of closure in carefully selected patients with probable paradoxical embolism.

This approach is aligned with contemporary position statements. The 2022 Society for Cardiovascular Angiography and Interventions PFO guidelines provide a conditional recommendation for closure in patients with systemic embolism after exclusion of alternative causes, even in the absence of prior stroke.^8^ Similarly, the British Cardiovascular Intervention Society supports consideration of closure in patients younger than 60 years with unexplained myocardial infarction or systemic embolism consistent with probable paradoxical embolism.^9^ Long-term follow-up after PFO closure in patients with paradoxical embolism suggests durable clinical efficacy, with infrequent recurrent ischemic events over 20 years, including recurrent stroke in fewer than 1% of patients.^10^

The key management decision was whether the PFO represented an incidental finding or a modifiable conduit for paradoxical embolism. The rationale for closure did not rest on the presence of a PFO alone, but on the overall diagnostic pattern supporting probable paradoxical coronary embolism. In this context, the heart team favored percutaneous closure over prolonged anticoagulation alone, acknowledging the absence of coronary-specific randomized data and the inability to establish causality definitively. This reflected a mechanism-informed secondary prevention strategy in a young patient for whom cumulative future embolic risk was an important consideration.

## Conclusions

This case highlights the importance of considering coronary embolism in patients with myocardial infarction and otherwise angiographically smooth coronary arteries, particularly when an abrupt distal vessel cut-off is identified without an atherosclerotic culprit lesion. Despite the absence of a demonstrable venous thromboembolic source, the overall diagnostic pattern supported probable PFO-mediated paradoxical coronary embolism. More broadly, this case illustrates how integrating multimodality imaging findings can refine plaque-free myocardial infarction from a descriptive presentation into a mechanism-informed diagnosis that guides secondary prevention.

**Table 1:** Equipment List

Procedure/Imaging Modality	Equipment
Coronary angiography	Terumo 5-F Introducer II (PQ); Terumo 6-F Glidesheath Slender sheath; Terumo 5-F Radial TIG catheter; 5-F Pig catheter; 5-F JR4 catheter
Transthoracic echocardiography	Philips EPIQ 7
Transesophageal echocardiography	Philips EPIQ 7; X8-2T transducer
PFO closure	Terumo 8-F × 10-cm sheath; Boston Scientific 5-F MPA2 catheter; 25-mm Amplatzer PFO occluder; Abbott Vascular 6-F ProGlide closure device

Equipment used for coronary angiography, transthoracic echocardiography, transesophageal echocardiography, and percutaneous patent foramen ovale closure.

PFO = patent foramen ovale; TIG = Tiger; JR = Judkins right; MPA = multipurpose angiographic.

## Funding Support and Author Disclosures

The authors have reported that they have no relationships relevant to the contents of this paper to disclose.
